# The Pathophysiological, Genetic, and Hormonal Changes in Preeclampsia: A Systematic Review of the Molecular Mechanisms

**DOI:** 10.3390/ijms25084532

**Published:** 2024-04-20

**Authors:** Yi-Ting Chiang, Kok-Min Seow, Kuo-Hu Chen

**Affiliations:** 1 Department of Medical Education, Taipei Tzu-Chi Hospital, The Buddhist Tzu-Chi Medical Foundation, Taipei 231, Taiwan; 107311109@gms.tcu.edu.tw; 2 Department of Obstetrics and Gynecology, Shin Kong Wu Ho-Su Memorial Hospital, Taipei 111, Taiwan; m002249@ms.skh.org.tw; 3 Department of Obstetrics and Gynecology, National Yang-Ming Chiao-Tung University, Taipei 112, Taiwan; 4 Department of Obstetrics and Gynecology, Taipei Tzu-Chi Hospital, The Buddhist Tzu-Chi Medical Foundation, Taipei 231, Taiwan; 5 School of Medicine, Tzu-Chi University, Hualien 970, Taiwan

**Keywords:** preeclampsia, VEGF, PlGF, sFlt-1, hormones, genetics

## Abstract

Preeclampsia, a serious complication of pregnancy, involves intricate molecular and cellular mechanisms. Fetal microchimerism, where fetal cells persist within maternal tissues and in circulation, acts as a mechanistic link between placental dysfunction and maternal complications in the two-stage model of preeclampsia. Hormones, complements, and cytokines play pivotal roles in the pathophysiology, influencing immune responses, arterial remodeling, and endothelial function. Also, soluble HLA-G, involved in maternal–fetal immune tolerance, is reduced in preeclampsia. Hypoxia-inducible factor 1-alpha (Hif-α) dysregulation leads to placental abnormalities and preeclampsia-like symptoms. Alterations in matrix metalloproteinases (MMPs), endothelins (ETs), chemokines, and cytokines contribute to defective trophoblast invasion, endothelial dysfunction, and inflammation. Preeclampsia’s genetic complexity includes circRNAs, miRNAs, and lncRNAs. CircRNA_06354 is linked to early-onset preeclampsia by influencing trophoblast invasion via the hsa-miR-92a-3p/VEGF-A pathway. The dysregulation of C19MC, especially miR-519d and miR-517-5p, affects trophoblast function. Additionally, lncRNAs like IGFBP1 and EGFR-AS1, along with protein-coding genes, impact trophoblast regulation and angiogenesis, influencing both preeclampsia and fetal growth. Besides aberrations in CD31+ cells, other potential biomarkers such as MMPs, soluble HLA-G, and hCG hold promise for predicting preeclampsia and its complications. Therapeutic interventions targeting factors such as peroxisome PPAR-γ and endothelin receptors show potential in mitigating preeclampsia-related complications. In conclusion, preeclampsia is a complex disorder with a multifactorial etiology and pathogenesis. Fetal microchimerism, hormones, complements, and cytokines contribute to placental and endothelial dysfunction with inflammation. Identifying novel biomarkers and therapeutic targets offers promise for early diagnosis and effective management, ultimately reducing maternal and fetal morbidity and mortality. However, further research is warranted to translate these findings into clinical practice and enhance outcomes for at-risk women.

## 1. Introduction

Among pregnancy-related complications, hypertensive disorders of pregnancy significantly contribute to maternal and perinatal mortality on a global scale. In Latin America and the Caribbean, hypertensive disorders are responsible for almost 26% of maternal deaths, whereas in Africa and Asia they contribute to 9% of deaths [1].

Preeclampsia (PE) is one of several hypertensive disorders of pregnancy and is defined by the International Society for the Study of Hypertension in Pregnancy (ISSHP) as gestational hypertension after 20 weeks’ gestation, accompanied by one or more of the following new-onset conditions: proteinuria, other maternal organ dysfunctions, or uteroplacental dysfunction. Maternal organ dysfunction includes acute kidney injury, liver involvement, neurological complications, and hematological complications [2,3].

Preeclampsia plays a serious and potentially life-threatening role, affecting both the antepartum and postpartum periods. Preeclampsia can deteriorate rapidly and without warning. Approximately 2% to 8% of pregnancies worldwide are affected by preeclampsia [1,4]. Not only does preeclampsia lead to poor maternal and fetal outcomes in pregnancy, but also adverse health outcomes for both mothers and babies in the long term. It is responsible for more than 500,000 fetal and neonatal deaths and 70,000 maternal deaths [4]. Furthermore, compared to women who have experienced a normal pregnancy, those suffering from preeclampsia have about double the risk of lifetime cardiovascular diseases, including cardiovascular death, coronary artery disease, heart failure, and stroke after adjusting for potential confounders. This risk is evident in the first 1–3 years of follow-up and remains significant until more than ten years of follow-up [5].

There are many intricacies when it comes to the etiology of preeclampsia. Preeclampsia has been considered a “disease of theories,” involving multiple pathological processes [2,3]. The true cellular and molecular mechanisms underlying preeclampsia remain largely unexplained, but are assumed to be a two-stage process of impaired uteroplacental perfusion with or without prior defective trophoblast invasion (Stage 1), followed by general endothelial dysfunction and vascular inflammation that lead to systemic organ damage (Stage 2) [6,7,8,9]. The defective trophoblast invasion leads to a hypoxic environment, causing increased oxidative stress, which characterizes subsequent placental pathologies and preeclampsia. In this pathway, the increased oxidative stress further worsens the inflammatory condition in these pregnancies [10]. Various forms of preeclampsia could be present, each following distinct pathophysiological pathways that contribute to maternal and fetal health risks [11,12]. The two primary subtypes frequently discussed are preeclampsia that occurs early in pregnancy (before 34 weeks of gestation), named early-onset preeclampsia, and that which develops later (at or after 34 weeks of gestation), named late-onset preeclampsia [12].

As mentioned above, preeclampsia and its associated complications have major impacts on maternal and fetal health. Therefore, the review aims to summarize existing studies in the literature to explore the underlying pathophysiological mechanisms of preeclampsia, as well as the roles and effects of genetics and hormones in preeclampsia.

## 2. Methods: Literature Review and Data Search

### Search Terms and Strategies in the Literature

**Figure 1:**
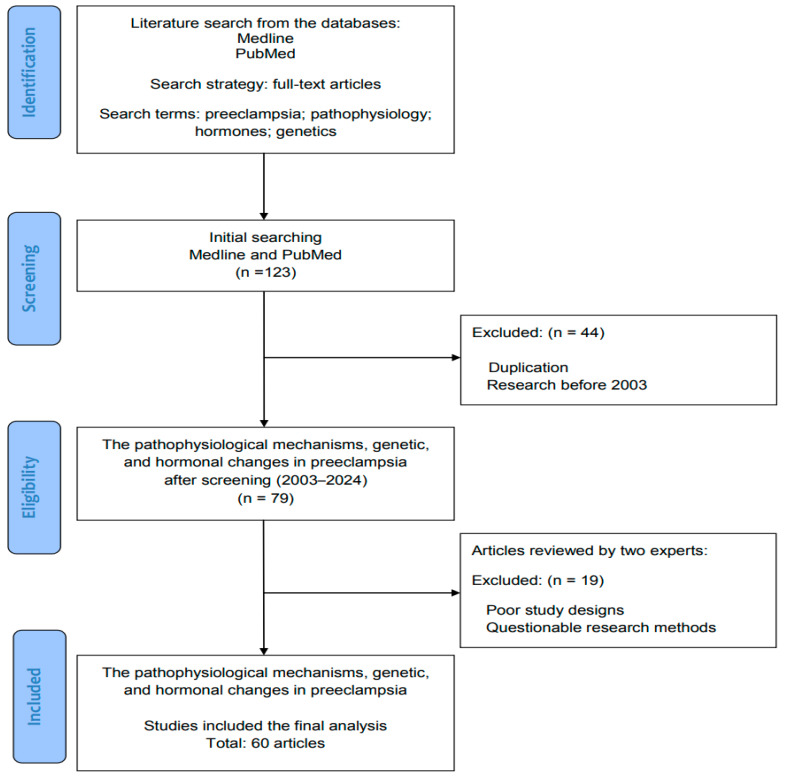
The flowchart for database searching, screening, selection, and inclusion of eligible articles from the literature.

The literature was searched to retrieve basic and clinical research that investigated the underlying molecular and cellular mechanisms of preeclampsia, along with the genetic, hormonal, and pathophysiological changes. Figure 1 illustrates the flowchart of database searching, screening, and the inclusion of the references that we collected from the literature. In the current review, all studies were solicited from the databases Medline and PubMed using the search terms “preeclampsia”, “pathophysiology”, “hormones”, and “genetics” for the research topic. For screening and selection in the second stage, only full-text articles were considered for inclusion in a subsequent analysis. In the next stage, duplicated articles were excluded. The articles published before 2003 were also excluded to ensure the novelty of this review. From a total of 123 articles identified in the screening process, 79 articles (2003–2024) met the criteria for further inclusion.

Hereafter, two experts in the field independently inspected the contents of the articles, including research designs and materials, and identified the eligible basic and clinical research for inclusion. The retrieved articles with poor research designs and questionable research methods were excluded in this stage. The discrepancies between two interacting experts were discussed to reach a consensus. According to the inclusion criteria (full-text articles; search terms “preeclampsia”, “pathophysiology”, “hormones”, and “genetics”; good quality) and exclusion criteria (duplicated articles; articles published before 2003; poor research designs and questionable research methods), all eligible articles were included in this review using the search terms and strategies (identification from the database, screening of the potential articles, selection of studies, and final inclusion). Finally, a total of 60 articles were identified for review from 123 articles solicited in the initial search.

## 3. The Pathophysiology and Placental Changes in Preeclampsia

### 3.1. The Two-Stage Model of Preeclampsia

Currently, the knowledge of preeclampsia encompasses nuanced distinctions between its early- and late-onset forms. Based on the limited understanding of preeclampsia, the revised “two-stage model” has become one of the most widely accepted theories regarding its formation.

**Figure 2:**
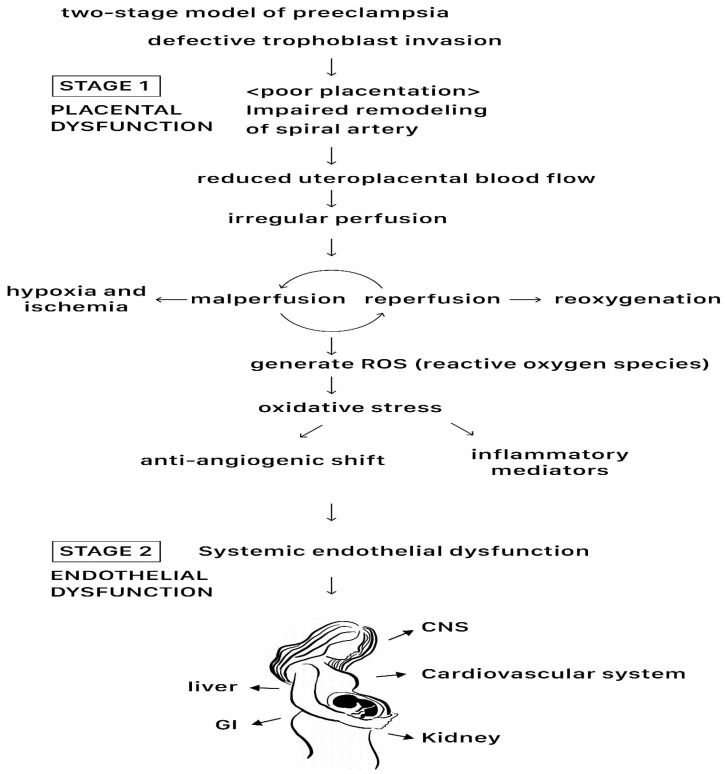
The two-stage model of preeclampsia, showing the process of initial defective trophoblast invasion and subsequent systemic endothelial dysfunction.

**Figure 3:**
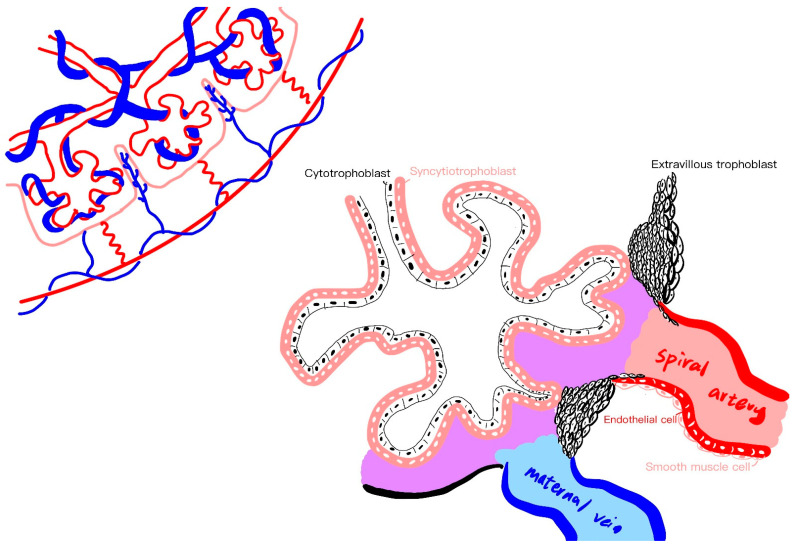
The vasculature and cells in the placenta.

In this model, the initial phase, termed the “placental stage”, implicates an inadequate remodeling of spiral arteries, culminating in compromised placental perfusion and ischemia. Progression to the second stage ensues as clinical maternal symptoms materialize [7,13]. Stage 1 centers on impaired uteroplacental perfusion, involving deficient placentation and spiral artery insufficiency, while Stage 2 highlights systemic endothelial dysfunction and vascular inflammation, eliciting a broader and more serious clinical response [7,9,11] (Figure 2). The vasculature and cells in the placenta are illustrated in Figure 3.

Stage 1 commences with reduced placental perfusion, often attributed to poor placentation and deficient spiral artery remodeling. Although these factors contribute significantly, maternal influences also play a pivotal role in instigating systemic pathophysiological changes [7,11,13]. Typically unfolding in the first trimester, Stage 1 coincides with the invasion of extravillous trophoblasts (EVTs) into the decidua, critical for uteroplacental perfusion and fetal blood supply [14]. This phase may initiate as early as before GA 8 weeks, with the establishment of uteroplacental circulation completed around GA 12 weeks, suggesting a timeline for Stage 1 of before GA 12 to 20 weeks [15].

EVT differentiation and invasion are orchestrated by an array of factors including cytokines, growth factors, chemokines, and cell adhesion molecules, serving as potential markers for the initial phase [7,11,14,15]. Defective trophoblast invasion and the inadequate transformation of the maternal uterine vasculature lead to a decreased maternal uterine blood flow and subsequent reactive oxygen species (ROS) formation (Figure 2), detectable through uterine artery Doppler studies. A high vessel resistance in early pregnancy indicates this phenomenon, with placental endothelial cells in women with high-resistance uterine arteries displaying a heightened susceptibility to TNFα-induced injury and apoptosis.

Conversely, in the normal trophoblast invasion process, uterine artery blood flow resistance decreases as placental development progresses, peaking in the first trimester [14]. An altered balance of antioxidant enzyme activity in hypoxic environments may affect placental tissues, albeit histopathology findings of the placenta are often nonspecific and not exclusive to preeclamptic pregnancies at this moment. These changes in the placenta can also be induced by other microscopic insults or toxins [16].

Stage 2 denotes a juncture where impaired uteroplacental perfusion intertwines with various maternal constitutional factors, manifesting in pathophysiological changes across multiple organ systems, indicative of an insufficient blood supply. Systemic endothelial dysfunction and injury underlie maternal clinical manifestations and are proven to be present in preeclamptic women (Figure 2) [6,7,8,11].

**Figure 4:**
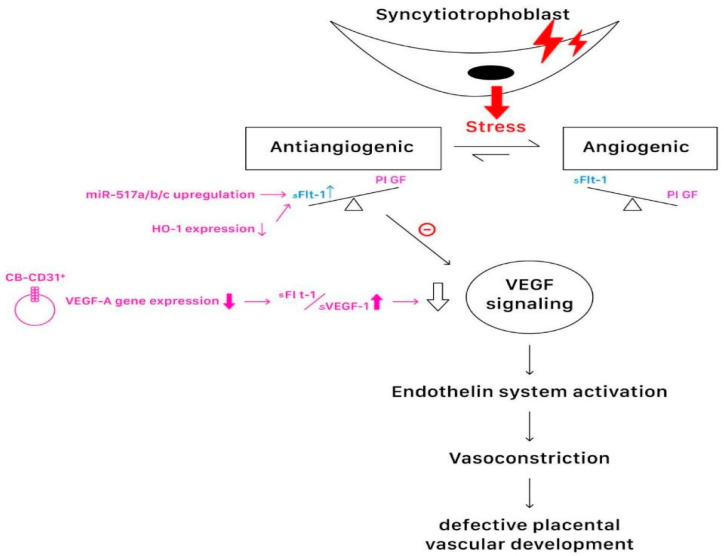
The linkage between Stage 1 and Stage 2, involving microparticles produced during syncytiotrophoblast stress, which include inflammatory mediators, vascular endothelial growth factor (VEGF), placental growth factor (PlGF), soluble fms-like tyrosine kinase 1 (sFlt-1), and oxidative stress accumulation.

The linkage between Stage 1 and Stage 2 is a subject of keen interest, with proposals attributing it to microparticles produced during syncytiotrophoblast apoptosis, inflammatory mediators, the renin–angiotensin system, vascular endothelial growth factor (VEGF), placental growth factor (PlGF), soluble fms-like tyrosine kinase 1 (sFlt-1), and oxidative stress accumulation (Figure 4) [8,9,17]. Artery compliance regulation during pregnancy involves numerous factors, with dysregulation contributing to hypertensive disorders such as preeclampsia. Changed circulating cytokines and growth factors may inhibit normal calcium signaling events, thus damaging cell-to-cell contacts of the endothelium and leading to endothelial dysfunction [18,19,20]. Noteworthy markers include endothelin-1 (ET-1), interleukin-8 (IL-8), ELAM, and endothelial leukocyte adhesion molecule-1 [21]. Reduced nitric oxide (NO) bioavailability, along with lowered levels of other vasodilators (prostacyclin and endothelium-derived hyperpolarizing factor), further compounds vascular dysfunction [22].

The involvement of the renin–angiotensin system (RAS) in preeclampsia’s pathogenesis is underscored by studies revealing increased angiotensin II type 1 (AT1) receptor expression in preeclamptic pregnancies. Circulating agonistic autoantibodies (AAs) targeting AT1 receptors have been described previously, which have the ability of crossing the placenta and entering the fetal circulation. AT1-AAs could induce calcium signaling changes and initiate events with resultant preeclampsia [23]. In one study, AT1-AA injections evoked preeclampsia in mice. Furthermore, AT1-AA might be associated with increased tumor necrosis factor α (TNF-α) and elevated anti-angiogenic factors such as soluble fms-like tyrosine kinase-1 (sFlt-1) (also named VEGFR-1) and endoglin, indirectly causing damage to the endothelium and target organs [24]. Oxidative stress, arising from an imbalance between ROS formation and antioxidant capacity, is implicated in hypertension and preeclampsia, accentuating the role of mitochondrial dysfunction and placental oxidative damage in maternal complications [25].

In addition, immature platelet fraction and thrombin generation play a critical role in preeclampsia. Throughout pregnancy, hemostatic changes lead to a state of hypercoagulability, elevating the risk of thrombosis. These changes are exacerbated in women with preeclampsia due to the abnormal activation of both the hemostatic and immune systems [1]. In gravidas complicated with preeclampsia, platelets are activated due to excessive thrombin generation, correlating with a uterine hemorrhage at the moment of syncytiotrophoblast implantation in the first trimester. Thus, this activation may contribute to an increased release of anti-angiogenic factors and the initiation of the complement system before the onset of clinical symptoms related to the syndrome. In practice, these thrombotic and anti-angiogenic factors could serve as biomarkers of preeclampsia. Consequently, exploring laboratory tests measuring the IPF (immature platelet fraction) and evaluating thrombin generation could potentially aid in the early diagnosis and management of preeclampsia [1].

### 3.2. Placental Findings in Preterm and Term Preeclampsia: The Pathological Changes in the Placenta

The anatomopathological characteristics of the placenta in early-onset preeclampsia are significantly different from those in late-onset preeclampsia. In cases of early-onset preeclampsia, large morphological changes include areas of hypoxia, villous infarctions, and placental hypoplasia; anatomopathological changes are most likely an attempt to ascertain adequate blood flow to the fetus [26].

After the Amsterdam Consensus, maternal vascular malperfusion (MVM) is conceptually deemed a critical morphological change during preeclampsia, and it can be found in the placental lesions with tissue ischemia, maternal vascular underperfusion, placental insufficiency, and Tenney–Parker changes. MVM can be categorized into two histological findings: partial and complete.

In cases of partial MVM, microscopic examinations reveal localized and accelerated villous maturation, which refers to areas within the placenta showing both underdeveloped regions with fewer villi and regions with increased villous clustering, elevated syncytial nodes, perivillous fibrin accumulation, and villous agglutination. In this type of MVM, there is a presence of smaller or less developed villi compared to those in a normal placenta of the same gestational age, indicating restricted blood flow due to the inadequate remodeling of uterine arteries and decidual arteriopathy, resulting in an uneven maternal blood supply [26].

In the case of complete MVM, microscopic analyses reveal villous infarctions. These affected areas indicate placental necrosis due to the complete loss of maternal blood flow. Depending on the age of the infarction, the trophoblasts show reduced nuclear basophilia, and accompanied findings include the destruction of lumens within the fetal vessels, stromal fibrosis, and the complete breakdown of trophoblasts in the fetal vessels, all of which result in a collapse of the intervillous space and the presence of fibrin around the villi [26].

### 3.3. The Roles of Medications in Preeclampsia: Assessment of the Proton Pump Inhibitors (PPIs) Esomeprazole Magnesium Hydrate and Trihydrate

Proton-pump inhibitors (PPIs) such as lansoprazole, rabeprazole, and esomeprazole are commonly used to alleviate symptomatic gastric acid reflux, and they have been shown to decrease the placental release of sFlt-1 and soluble endoglin in vitro. Focusing on the roles of PPIs as pathophysiological markers in preeclampsia in preclinical human models of disease, studies have revealed that PPIs can mediate multiple aspects of the pathophysiology of preeclampsia. Among several PPIs, esomeprazole magnesium hydrate is most effective in enhancing vascular relaxation and can reduce the levels of key factors associated with preeclampsia, such as sFlt-1, and thus, can improve endothelial dysfunction [27].

In the in vitro studies comparing esomeprazole magnesium hydrate with its hydration isomer, esomeprazole magnesium trihydrate, the former appears more efficacious in mitigating pathogenic actions than the latter. In the preclinical models of preeclampsia, both drugs could mitigate the expressions of endothelial dysfunction markers, including vascular cell adhesion molecule-1 and endothelin-1, and reduce the secretion and expression of sFlt-1 (anti-angiogenic factor) from primary cytotrophoblasts, but only esomeprazole magnesium hydrate reduced sFlt-1 secretion from primary human umbilical vein endothelial cells [27].

A double-blind, randomized controlled trial compared the administration of 40 mg of esomeprazole daily to a placebo in women with preterm preeclampsia between gestational ages 26 weeks + 0 days and 31 weeks + 6 days. Higher doses, twice-daily dosing, or intravenous administration have been suggested for potential efficacy; however, further evaluation is necessary [28,29]. On the other hand, the lowest effective dose of PPIs for short-term use during pregnancy is not studied well.

While preclinical studies have suggested that PPIs may have preventive effects on preeclampsia, certain studies have raised concerns regarding the association between PPI use and the risk of developing preeclampsia [30,31,32,33]. A meta-analysis revealed a slight elevation in the risk of preeclampsia among pregnant women using PPIs. Moreover, there is insufficient evidence to support the assertion that PPI administration definitely reduces the risk of either preeclampsia or preterm preeclampsia [33].

Currently, the safety of PPIs during pregnancy has not been extensively studied. Limited human research and animal studies provide the majority of the evidence. As a result, it is unclear what risks the fetus may face. Some studies have concluded that intrauterine exposure to PPIs during the first trimester of pregnancy is not associated with an increased risk of teratogenicity [34,35,36]. Nevertheless, some studies have found a possible link between using PPIs during pregnancy and specific birth problems in spite of no direct evidence that PPIs cause birth defects.

The possible benefits and hazards of using PPIs during pregnancy should be carefully discussed with pregnant women before making a choice. In certain instances, the advantages of using PPIs to treat certain diseases might exceed possible hazards to the developing fetus. If there is a major concern regarding the risks of PPIs during pregnancy, alternative therapies that present fewer dangers during pregnancy, including antacids or H2-receptor antagonists, can be considered depending on the severity of the condition.

## 4. Genetic and Hormonal Changes in Preeclampsia

### 4.1. The Genetic Nature of Preeclampsia

#### 4.1.1. Circular RNA (CircRNA)

CircRNA_06354 might promote early-onset preeclampsia in humans via the actions of hsa-miR-92a-3p/vascular endothelial growth factor-A (VEGF-A). A study pointed out that the circRNA_06354/hsa-miR-92a-3p/VEGF-A signaling pathway is involved in the progression of early-onset preeclampsia (EOPE) resulting from the aberrant expression of these molecules, which could lead to the dysregulation of trophoblastic cells invading the spiral arteries or even moving toward the superficial myometrium and could ultimately lead to the onset of EOPE [37].

The expression levels of circRNA_06354 in the placenta of women with EOPE differ from those in their blood. In EOPE, shallow trophoblastic cell invasion into the spiral arteries, or even toward the myometrium, is dependent on the expression of circRNA_06354 in the placental cells rather than in the blood cells. The overexpression of circRNA_06354 has been found to inhibit trophoblastic cell invasion, migration, and tube formation toward the endometrium during the initiation of EOPE. Moreover, the observed increased levels of circRNA_06354 in EOPE placentas, but not in EOPE blood, support the involvement of circRNA_06354 in the progression of EOPE [37].

The subcellular localization of circRNA_06354 in EOPE placentas compared to that in preterm or term placentas has revealed that the syncytiotrophoblast, rather than cytotrophoblast, has the ability to exchange nutrients or constituents with blood cells. This finding further strengthens the idea that lower levels of circRNA_06354 in EOPE blood cells are due to the localization of circRNA_06354 in the cytotrophoblasts of EOPE placentas [37].

CircRNAs act as microRNA (miRNA) sponges, resulting in a ceRNA network that participates in the pathogenesis of EOPE. CircRNA_06354 might sponge hsa-miR-92a-3p, thereby targeting VEGF-A in the pathogenesis of EOPE. Hsa-miR-92a-3p has been associated with the occurrence of hypertension. In hypoxic conditions such as in EOPE, endothelial cells in the placenta are activated, resulting in increased VEGF-A production. Endothelial cell activation is considered as an initiating factor in the progression of preeclampsia. Consequently, the increased expression of VEGF-A in EOPE leads to poor placentation, initiating the development of EOPE [37].

#### 4.1.2. microRNA (miRNA)

Chromosome 19 microRNA cluster (C19MC), representing one of the largest human miRNA clusters, is expressed almost exclusively in the placenta. The expression of the C19MC is detected as early as 5 weeks of pregnancy and markedly increases in placental trophoblasts from the first to the third trimester. Different types of C19MCs have various functions, and certain types are required for adequate placentation and selectively attenuate migration of human trophoblasts [7,38]. The dysregulation of C19MC has an impact on trophoblast differentiation, invasion, and angiogenesis [39].

In detail, miR-519d, a member of the C19MC, regulates the invasive phenotype of the extravillous trophoblast (EVT), consequently suppressing trophoblast invasion and migration. Mir-515-5p is upregulated in the placenta with PE, and its overexpression leads to an inhibition of syncytiotrophoblast differentiation, targeting key molecules in the process [7,38]. MiR-517-5p is highly expressed in the placenta in PE, leading to a decrease in the proliferative and invasive abilities of cells from the human choriocarcinoma cell line. The upregulation of miR-517a/b/c in the primary EVTs in the first trimester results in decreased trophoblast invasion and an increased release of the anti-angiogenic protein sFlt1 (Figure 4). The aberrant upregulation of miR-518b in the preeclamptic placenta may contribute to excessive trophoblast proliferation. The hypoxic condition during early placentation reduces C19MC expression and releases the inhibitor of epithelial-to-mesenchymal transition genes, leading to the acquisition of migratory and invasive characteristics of the EVTs [7,38]. Strub et al. showed that C19MC miRNA members could be involved in aberrant angiogenesis in infantile hemangioma, and the finding goes in line with the possible role that C19MC miRNA members may play in angiogenesis during pregnancy [39].

C19MC-derived miRNAs are secreted from primary human trophoblasts and released to maternal circulation as exosome vesicles, which can influence maternal immune cells and have a major impact in immune tolerance during pregnancy and defense against viral infection [14,40]. Therefore, it is possible that these exosomes, carrying a potentially aberrant miRNA repertoire, could severely influence maternal immune cells, leading to their dysfunction and, in that way, contributing to the maternal systemic inflammatory response and the development and progression of PE [7,38]. Additionally, circulating miRNAs could serve as unique biomarkers for monitoring trophoblast and placental function, both as predictive and diagnostic markers of the disease [40]. However, there is still minimal overlap between the individual miRNAs identified as potential biomarkers within the studies [39].

#### 4.1.3. Long Non-Coding RNA (lncRNA)

Another potential biomarker of PE is long non-coding RNAs (lncRNAs). Multiple lncRNAs related to PE are identified and involved in different stages of PE individually, which indicates that different pathophysiological mechanisms are driving early-onset and late-onset PE [41]. The upregulation and downregulation of specific lncRNAs can impact critical mechanisms in the development of PE and lead to changes in trophoblast proliferation, invasion, migration, and apoptosis [41]. Despite the investigation of several circulating lncRNAs (lncRNAs BC030099, AF085938, G36948, and AK002210) as potential biomarkers for PE, their validity is questioned due to their low abundance in circulation, leading to challenges in quantification, as well as unstandardized strategies in normalization [39].

Due to the extensive involvement of C19MC-derived miRNAs in the pathogenesis of PE, their detection in maternal circulation early in pregnancy and their exclusive placenta-specific pattern of expression signify their potential as promising prognostic and diagnostic tools for PE [39].

IncRNAs have recently gained prominence in placental research because of their involvement in pregnancy complications, as an altered expression of several lncRNAs has been associated with various placental disorders, positioning them as potential regulators of multiple molecular pathways implicated in the pathogenesis of placental diseases [41]. A study further compared the placental gene expression profiles including mRNA and lncRNAs among pregnant women who had experienced PE, intrauterine growth retardation (IUGR), or both [42].

There exists significant over-expression in nine selected protein-coding genes, including *IGFBP1* (Insulin-Like Growth Factor Binding Protein 1), *FGC* (Fibrinogen Gamma Chain), *FBXO2* (F-Box Protein 2), *CPEB1*, *CHST2*, *CD40LG*, *CATSPER1*, *CABYR*, and *STAR* (Steroidogenic acute regulatory protein) and the lncRNA *EGFR-AS1* in IUGR samples compared to the normal development group [42].

STAR plays a pivotal role as a crucial regulator, controlling the rate of steroid production and cholesterol intracellular trafficking within steroidogenic tissues. The expression of genes such as IGFBP1 and PRL, as well as other genes involved in the p13-akt signaling pathway like GNB5 and GNG4, highlights the involvement of these pathways in placental ischemia. This protein-encoding gene is mainly associated with trophoblast implantation and invasion. Lowly expressed *IGFBP* during the first trimester of pregnancy has been implicated in implantation failure and impaired placentation, leading to the placental insufficiency observed in both conditions. Additionally, robust evidence demonstrates the high expression of this protein in term placentas from pregnancies complicated with PE or IUGR. Nevertheless, the roles of *IGFBPs* as potential biomarkers are discrepant among studies [42].

#### 4.1.4. CD Genes and Intrauterine Growth Retardation (IUGR)

The dysregulation of genes shared between PE and IUGR involves several immunologic responses such as cytokine-mediated signaling pathways, inflammatory responses, and immune regulation, indicating excessive inflammatory upregulation in both PE and IUGR. These genes include CD40L, TNFRSF8, IL1R2, LRRC15, and ZNF683, as well as genes related to the JACK-STAT cascade such as PRL and OLAH [42].

Moreover, the samples of pregnancies complicated with PE show upregulation for *CABYR*, *CATSPER1*, and *EGFR-AS1*. It is also observed that *CPEB1* and *IGFBP1* are both upregulated in the PE-IUGR group compared to the normal pregnancy group. *EGFR-AS1* has been reported as an important target in the pathophysiology, as its overexpression promotes cell proliferation. *EGFR-AS1* knockdown is associated with the decreased expression of p-JAK and p-STAT; thus, the observation indicates that this lncRNA regulates the JAK-STAT signaling pathway. Furthermore, this study notes that the upregulation of this pathway not only occurs in PE but also in IUGR [42].

The samples of pregnancies with IUGR exhibit the most significant transcript variations, displaying the downregulation of genes involved in neuroactive ligand–receptor interaction, fatty acid biosynthesis, and pathways involving nitric oxide synthase 3 (NOS3) activity, which encompass arginine biosynthesis and metabolism, angiogenesis, as well as the VEGF signaling pathway. In particular, endothelial nitric oxide synthase 3 (eNOS3) plays a role in placental angiogenesis and vasculogenesis, displaying high expression levels during normal embryonic and fetal development [43]. A low expression of eNOS3 correlates directly with a lower nitric oxide availability, impairing trophoblast invasion and consequently resulting in a reduced uteroplacental blood flow and oxygen levels observed in cases of IUGR. Moreover, it has been suggested that a defective eNOS function and elevated oxidative stress might contribute to early endothelial dysfunction in individuals with IUGR, potentially leading to development of hypertensive disorders [42,43].

A comparative analysis on the placental transcriptome performed in a study clearly indicates that PE and IUGR share altered placental pathophysiological pathways, which are mainly associated with immunological processes, cholesterol, and protein metabolism [14,42]. In preceding research, severe PE is shown to be associated with the reduced expression of angiogenic genes and with disturbances in the control of inflammatory genes within the placenta [43]. The impact of PE on hematopoietic and vascular cells originating from the developing fetus remains unclear. However, there are reports indicating that PE affects fetal hematopoiesis not solely due to restricted growth but as a direct consequence of PE itself. Preeclamptic cord blood (CB) displays a decrease in the quantity of hematopoietic stem cells alongside a simultaneous reduction in endothelial colony-forming cells [44].

CD31+ mononuclear cells (MNCs) derived from human peripheral blood represent a broad spectrum of circulating cells which possess angiogenic and vasculogenic properties. CD31+ cells have emerged as a biomarker representing an angio-vasculogenic population among MNCs. A study aimed to identify abnormalities in subpopulations of CB-MNCs from PE patients through a flow cytometry analysis, and further investigated alterations in the expression of genes accounting for abnormal blood vessel formation and inflammation associated with PE placentas [44]. The study demonstrated that in severe PE, but not mild PE, there is a decrease in various subpopulations of CB- MNCs, including CD31+ cells, along with a reduction in VEGF-A gene expression and increased inflammatory gene expression (Figure 4). CD31+ cells, CD14+ cells, CD11b+ cells, and CD3+ cells rather than others were significantly reduced in the CB-MNCs of severe PE patients, among which CD31+ cells and CD3+ cells showed the most notable changes. Moreover, CD14+ cells, which yielded cells with an endothelial characteristic and a functional role in neovascularization, were decreased in the MNCs and the CD31+ cell fraction from severe PE patients. Thus, a reduced number of CD14+ cells in cord blood further suggests the impaired angio-vasculogenic function of CB-MNCs that is closely associated with severe PE [44].

The mRNA expression of the major angiogenic factor VEGF-A is substantially reduced in both CB-MNCs and CB-CD31+ cells from severe PE patients. The results correspond well with prior studies demonstrating the elevation of sFlt-1/sVEGF-1 in the maternal circulation and placenta, which are inhibitors of the VEGF signaling pathway (Figure 4). The difference in angiogenic gene expression is more significant in CB-CD31+ cells than those observed in CB-MNCs, suggesting the importance of CD31+ cells in reflecting the changes in angiogenic factors in severe PE. On the other hand, the crucial inflammatory cytokines IL-1 and CXCL12 are increased in CB-MNCs and CB-CD31+ cells [44]. In conclusion, the results support the pathophysiology of defective placental vascular development and suggest that CB-CD31+ cells and their gene expression patterns have pathophysiological significance and the potential to serve as a biomarker for severe PE [44].

### 4.2. The Molecular and Cellular Effects of Hormones, Complements, and Cytokines in Preeclampsia

#### 4.2.1. Fetal Microchimerism in the Two-Stage Model of Preeclampsia

During gestation, fetal cells traverse the placental barrier, enduring within maternal tissues and circulation, a phenomenon known as fetal microchimerism. In the context of the two-stage model of preeclampsia, syncytiotrophoblast stress acts as a shared pathophysiological pathway underlying both maternal and fetal clinical manifestations of preeclampsia. Excessive syncytiotrophoblast stress triggers the anti-angiogenic shift involving placental factors: soluble fms-like tyrosine kinase-1 (sFlt-1) and placental growth factor (PlGF) [8,9]. A correlation between fetal microchimerism and these factors suggests an association between fetal microchimerism and placental dysfunction [1,39].

Beyond recognized placental stress markers (such as an imbalance in anti-angiogenic proteins) in the maternal circulation, fetal cells contribute to the progression from Stage 1 to Stage 2 preeclampsia. Fetal microchimerism serves as a mechanistic link between Stage 1 placental dysfunction and Stage 2 maternal cardiovascular inflammation and endothelial dysfunction (Figure 2) [1,39].

Preeclampsia affects cardiovascular health by exacerbating pre-existing conditions or instigating new ones via inducing placental dysfunction, resulting in the release of anti-angiogenic signaling molecules [45,46,47,48,49,50] and inflammatory mediators, and ultimately resulting in systemic endothelial dysfunction. Fetal microchimerism emerges as a plausible candidate for an inflammatory factor that potentially contributes to this pathophysiological cascade [1].

Fetal cell trafficking is upregulated by and further contributes to systemic maternal vascular inflammation, therefore causing endothelial dysfunction and raising blood pressure. This association between fetal microchimerism and the Stage 2 maternal response to placental dysfunction further implicates its potential role as a mediator in fetal–maternal crosstalk during preeclampsia [39].

#### 4.2.2. The Roles of Hormones, Complements, and Cytokines in Preeclampsia

A study identified the pathophysiological pathways involved in EOPE through a proteomics analysis. Five significantly enriched pathways were noted, whereby the activation of the complement and coagulation cascades occurred on top, including five proteins (coagulation factor IX, complement C3, von Willebrand factor (VWF), kininogen-1, and complement C2). To validate these findings, endothelial cells were exposed to activated plasma from early-onset severe preeclampsia. The results showed significantly higher deposits of the C5b-9 complement complex and VWF compared to controls. This reflects the activation of the complement and coagulation cascades in these patients and its effect on endothelial cells [51].

Soluble HLA-G (sHLA-G), an alternative splicing of HLA-G, is expressed by trophoblasts, immune cells, and other cells in various tissues. By activating receptors on immune cells, sHLA-G can mitigate maternal immune responses against fetal cells. Low sHLA-G levels in the maternal circulation are seen in all trimesters in pregnancies complicated by PE. The immunotolerance fostered by sHLA-G might facilitate the infiltration of EVTs into uteroplacental spiral arteries. Impaired infiltration could disrupt arterial remodeling, leading to an irregular and incomplete placental perfusion and subsequent stress on the syncytiotrophoblast. A study investigating sHLA-G levels in PE patients during pregnancy and postpartum periods revealed a negative correlation between circulating maternal sHLA-G and both early-onset PE and LOPE during pregnancy, displaying lower levels of sHLA-G compared to normotensive controls. Additionally, circulating sHLA-G was elevated in non-pregnant women until 1 or 3 years after EOPE. These findings suggest an excessively activated immune system in pregnant women with PE and may provide an immunological linkage to the epidemiological well-known increased risk of future cardiovascular disease in these women. In conclusion, sHLA-G plays a crucial role as a tolerogenic signaling molecule in placental function [52].

Hypoxia-inducible factor 1 alpha (Hif-α) serves as a principal regulator of trophoblast differentiation and plays a crucial role in oxygen homeostasis, which is necessary for placentogenesis [53]. According to in vivo research focusing on mouse placental development, prolonged Hif-α expression results in a significant decrease in fetal birth weight and substantial physiological alterations in placental differentiation, leading to pregnancy-associated disorders such as PE and/or fetal growth restriction. These alterations include reduced branching morphogenesis, alterations in maternal and fetal blood spaces, and failure to remodel the maternal spiral arteries [54].

The inactivation of Hif-α is essential for trophoblast differentiation, the invasion of giant cells, and the remodeling of spiral arteries in the phase of invasive trophoblast giant cells reaching the maternal spiral arteries in order to meet the increased demand of blood flow and oxygen for the developing placenta and fetus [53]. The lack of spiral artery remodeling would further generate a subsequent hypoxic environment in the junctional zone and labyrinth, and the end result would be the development of preeclampsia-like symptoms [54].

Alongside high maternal blood pressure, maternal glomeruloendotheliosis stands as a classic hallmark of PE. The research reveals significant increases in glomerular pathological damage, total urinary proteins, and the urinary albumin/creatinine ratio in response to Hif-α effects [53]. These findings indicate glomerular damage and dysfunction, suggesting that the prolonged presence of Hif-α, particularly in placental trophoblasts, promotes kidney damage and leads to proteinuria, consistent with a preeclamptic phenotype [54].

There is a pathophysiological involvement of HLA antibodies in PE. In the analysis of sera of women with uneventful pregnancies, PE, and gestational diabetes mellitus (GDM), two thirds of the women with an uneventful pregnancy or GDM were HLA- and MIC-A-antibody positive in gestational weeks 11 to 13, with a modest increase towards the end of pregnancy. However, women with PE showed an inverse kinetic: 90% were HLA-antibody positive in gestational weeks 11 to 13, and only 10% showed HLA reactivities at the end of the pregnancy. The HLA antibody binding strength was more pronounced in gestational weeks 14 to 17 in patients with PE compared with women with uneventful pregnancies, and this was able to predict PE with an AUC of 0.80. The assessment of HLA antibody levels during early pregnancy could serve as a valuable diagnostic tool to increase awareness among women at risk of developing PE [55].

**Figure 5:**
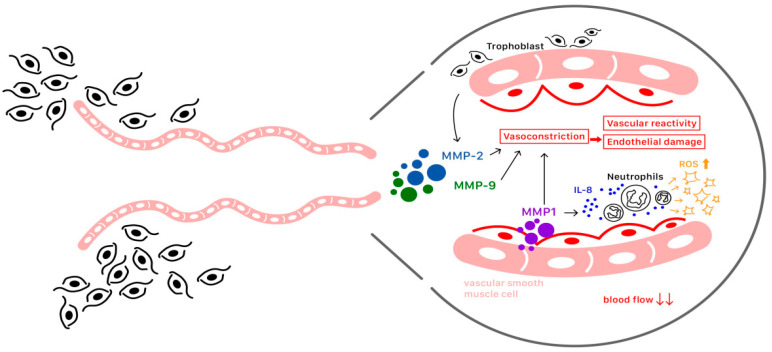
The roles of matrix metalloproteinases (MMPs) during trophoblast invasion, showing their effects on vasoconstriction, the subsequent generation of reactive oxygen species (ROS), and endothelial damage.

Matrix metalloproteinases (MMPs), which act as proteases, are expressed by trophoblast cells. MMPs and their endogenous inhibitors (TIMPs) are involved in trophoblast invasion into the uterine wall. The expression of MMP-2 upsurges in the early stages of gestation followed by a subsequent decline, while MMP-9 shows an ascending trend and remains predominant throughout the entirety of pregnancy. As MMP-2 is associated with implantation, MMP-9 is related to invasion. Alterations in the levels and activities of several MMPs and TIMPs contribute to defective trophoblast invasion and endothelial dysfunction, both of which are pathological features of PE (Figure 5). Villous samples from preeclamptic patients exhibit low concentrations of MMP-2 and MMP-9 due to the increased methylation of these enzymes, resulting in gene silencing. Consequently, the regulation of physiological trophoblast implantation and invasion is affected in pregnancies complicated with PE [56].

Additionally, apart from their impact on placental dysfunction, MMPs play a major role in vascular remodeling and in mediating vascular reactivity. Various MMPs, including MMP-1, MMP-2, and MMP-9, induce vasoconstriction, alterations in vascular reactivity, and endothelial damage, contributing to vascular dysfunction that develops during the late stage of PE. A high level of MMP-1 in the vasculature plays a role in the breakdown of vascular collagen, potentially contributing to the edema and proteinuria in patients with PE. Furthermore, MMP-1, released by vascular smooth muscle cells, stimulates the secretion of interleukin-8, promoting the recruitment of activated neutrophils in women affected by PE. This recruitment leads to the subsequent generation of reactive oxygen species (ROS) [56] (Figure 5).

As MMPs are a key factor in the pathophysiology of PE, they have been proposed as potential biomarkers for PE. Several studies have investigated various MMPs—such as MMP-1, MMP-2, MMP-9, and MMP-14—as diagnostic biomarkers. Additionally, recent epigenetic studies have focused on miRNA associated with the expression of MMP-2 and MMP-9. However, further clinical studies are necessary to validate these potential biomarkers for diagnosing PE [56].

Endothelins (ET)s, known as potent vasoconstrictors, are secreted by vascular endothelial cells and placental syncytiotrophoblasts. Elevated levels of sFlt-1 in preeclampsia result in angiogenic imbalance and are associated with the activation of the endothelin system in response to VEGF inhibition [8,45,46,47,48,49,50]. In an experimental preeclampsia model, the reduced uterine perfusion pressure model, an increase in blood pressure and the development of proteinuria were observed in rats after the administration of sunitinib, leading to two- to three-fold rises in circulating ET-1 levels. This outcome was also observed in swine. Therefore, the blockers, which can bind to endothelial receptors to inhibit their actions, have the potential to serve as therapeutic options. However, currently available endothelial receptor blockers are teratogenic. Nevertheless, the activated endothelin system plays a key role in the pathogenesis of PE [57].

A systematic review and meta-analysis pointed out that flow-mediated dilation, as an indicator of endothelial function, is lower in the women with PE both preceding the onset of preeclampsia, during the time of active disease, and persisting for three years postpartum [58].

CXCL3, a member of the chemokine family, affects trophoblast invasion and potentially plays a role in the pathophysiology of severe PE. Elevated plasma levels of CXCL3 and a reduced placental expression of CXCL3 were observed in severe PE, suggesting its involvement in shallow implantation [59].

As sFlt-1 and placental growth factor (PlGF) are both crucial components of the VEGF system in placental angiogenesis, an imbalance of these angiogenic factors is related to the development of PE [8,9,45,46,47,48,49,50]. Given human chorionic gonadotropin (hCG)’s role in regulating VEGFs, it is likely that hCG participates in the angiogenic processes in PE. A cohort study focused on the role of hCG in PE and its interaction with sFlt-1 and PlGF in the pathophysiology of PE. In the first trimester, high levels of hCG were associated with a reduced risk of preterm PE, contrasting with the increased risk observed with high hCG levels in the second trimester. Also, third-trimester high concentrations of hCG were linked to an increased risk of term PE. Additionally, high levels of hCG combined with low levels of PIGF in the second trimester conferred a very high risk of preterm PE [60]. Based on these findings, hCG and PIGF levels in different stages of pregnancy can be used as potential biomarkers for PE.

Women with a prior history of PE exhibit a higher susceptibility to subsequent cardiovascular disease. An analysis of the Preeclampsia Risk EValuation in FEMales (PREVFEM) cohort study investigated eight different cardiovascular biomarkers. The result showed that women 10 years after PE had higher levels of soluble endothelial selectin (SE selectin) and pregnancy-associated plasma protein A (PAPPA), potentially contributing to following cardiovascular events in the futures of post-PE women. Lower levels of ApoB were also observed, which may suggest its role in protecting against future cardiovascular events for women post-PE [61].

Microvesicles (MVs) are vesicles shed from cell membranes and involved in inflammation, apoptosis, and angiogenesis throughout pregnancy. As their content and characteristics may reflect the pathophysiological state of women with gestational vascular complications (GVCs), including gestational hypertension and preeclampsia/toxemia, a study analyzed their effects on endothelial cells and trophoblasts at early and term stages. Microvesicles from healthy pregnant women enhance endothelial cell functions, such as reducing apoptosis, stimulating migration, and promoting tube formation, while those from women with GVCs inhibit these processes. During early gestation, MVs mitigate trophoblast apoptosis while facilitating migration. However, this MV-mediated impact on early-stage trophoblast is absent in women with GVCs. Conversely, MVs in women with GVCs were observed to induce apoptosis in term trophoblast cells [62].

In pregnancies complicated by PE and IUGR, there is an observed upregulation of key transcriptional regulators of the hypoxic response including hif1α and p53, as well as their downstream target genes. Also, circulating cell-free fetal RNA was extracted from maternal plasma. Moreover, the result showed a high frequency of p21 and the roles of these hif1α-positive expressions in hypoxic pregnancies in comparison to normal pregnancies, supporting two items as possible surrogate markers in hypoxia and common complications of pregnancy [63].

Adhesion molecules play a key role and participate in the inflammatory cascade, regulating the adherence of leukocytes to endothelial cells and the migration of leukocytes into perivascular tissue. A study researching the function of adhesion molecules revealed that soluble E-selectin in women who subsequently developed preeclampsia was higher at 12–16 weeks and at 28 weeks than in those with uncomplicated pregnancies serving as controls. Thus, elevated soluble E-selectin levels may suggest endothelial cell activation and damage [64].

In some women who develop severe preeclampsia, the decreased placental expression of peroxisome proliferator-activated receptor (PPAR)-γ activators has been observed. PPAR-γ is a ligand-activated transcription factor expressed in trophoblasts. The activation of PPAR agonists through the upregulation of heme oxygenase 1 (HO-1), an antioxidant enzyme that negatively regulates sFlt-1 expression, has been demonstrated to mediate beneficial effects [65]. A study examined the effect of rosiglitazone, a selective PPAR-γ agonist, in the reduced uterine perfusion pressure (RUPP) rat. The results showed that the administration of rosiglitazone mitigated hypertension, enhanced vascular function, and decreased the elevated microalbumin/creatinine ratio in rats subjected to RUPP. Furthermore, these favorable outcomes were nullified in the presence of the HO-1 inhibitor, with the exception of the microalbumin/creatinine ratio [65]. Therefore, these results indicated that the PPAR-γ agonist prevented the development of several of the pathophysiological characteristics associated with the RUPP model of PE through the HO-1-dependent pathway [66].

Another study analyzed the endothelial function using a reactive hyperemia peripheral arterial tonometry (PAT) device and examined circulating biomarkers associated with lipid metabolism, angiogenesis, and inflammation in paired mothers and offspring 5 to 8 years after delivery. Maternal sFlt1 and high-sensitivity C-reactive protein levels remained elevated in women with a history of PE from delivery to 5 to 8 years postpartum. Notably, the endothelial function was significantly reduced in both mothers and children after PE when combined with a small-for-gestational-age (SGA) infant compared with mothers and children after pregnancies without an SGA infant. The dysfunction of endothelial cells may represent the mechanistic link between PE and a subsequent risk of cardiovascular disease [67].

Mediating leukocyte migration and adhesion, CX3CL1/fractalkine (CX3CL1) is a chemokine that is synthesized by endothelial cells and activated by proinflammatory cytokines. CX3CL1 and its receptor, CX3CR1, are expressed on invading human trophoblasts and are associated with uteroplacental vascularization. V249I and T280M polymorphisms of CX3CR1 protein form a common I_249_M_280_ and are related to a reduced risk of acute coronary events and atherosclerosis and with a lowered endothelial reactivity. As women with a history of PE predispose to future cardiovascular complications after delivery, a study attempted to explore the association between V249I and T280M polymorphisms of CX3CR1, PE, and endothelial dysfunction. Despite several pathophysiological similarities and common risk factors between atherosclerosis and PE, the results showed no differences in the genotype or haplotype frequencies between patients with PE and with normal pregnancies, suggesting that maternal CX3CR1, V249I, and T280M polymorphisms do not increase susceptibility to PE [68].

Invasive trophoblasts express the low polymorphic HLA-C, HLA-E, and the unique HLA-G rather than the highly polymorphic molecules HLA-A and HLA-B. HLA-G potentially contributes to the mechanism, enabling trophoblast invasion while evading attacks from the predominant lymphocyte population, including decidual NK cells. Decreased HLA-G RNA and protein levels have been found in a subset of preeclampsia patients. Trophoblasts with defective HLA-G expression are susceptible to attack by decidual NK cells, resulting in the poor remodeling of spiral arteries. Nevertheless, the interaction between decidual NK cells and trophoblasts actually benefits appropriate placental bed differentiation and development. Decidual NK cells produce chemokines, which attract trophoblasts to express chemokine receptors (CXCR1 and CXCR3) and produce factors active in angiogenesis, affecting the modification of vessels in the placental bed. These cytokines, growth factors, and angiogenic factors [45,46,47,48,49,50] secreted by decidual NK cells to the maternal–fetal interface are important in attracting trophoblasts to the decidua and in promoting the remodeling of the spiral arteries [69].

The mutual relationship among AT_1_-AAs, cytokines, and angiogenic factors are listed in Table 1. Women with preeclampsia possess angiotensin receptor agonistic autoantibodies (AT_1_-AAs) that bind to and activate the AT_1_ angiotensin receptor on various cells, thus eliciting multiple biological responses relevant to the pathophysiology of preeclampsia [70].

Several biochemical factors have been researched to disclose their relevance to PE. The platelet count and platelet aggregation ability are lower in preeclamptic women, indicating that both the number and function are altered. Furthermore, a study observed an association between impairment in platelet responsiveness and higher plasma levels of transforming growth factor beta (TGF-β_1_) [71] in preeclamptic women. Hence, TGF-β_1_ may participate in the pathophysiological events of preeclampsia that are dependent on platelet activation [72]. Moreover, a high concentration of inositol phosphoglycans (IPGs), a kind of phospholipid-derived putative second messenger of insulin, has been identified in human preeclamptic placentas, urine, and amniotic fluid. IPGs have been involved in the pathophysiology of preeclampsia. A study analyzed urine bioactive IPG P-type (P-IPG) in women with PE. P-IPG is remarkably high, as its level increases a few weeks before the onset of clinically diagnosed PE and begins to decrease after delivery [73]. Another study revealed that a high level of TNF-α is associated with an increased risk of PE in the third trimester [74].

## 5. Discussion

Preeclampsia remains a significant challenge in obstetrics, characterized by hypertension, proteinuria, and hematologic and systemic complications after 20 weeks of gestation. Understanding the molecular and cellular mechanisms underlying this condition is essential for improving maternal and fetal outcomes. Fetal microchimerism, the persistence of fetal cells within maternal tissues and circulation, is an emerging area of interest in the pathogenesis of preeclampsia. The two-stage model proposes that fetal microchimerism serves as a bridge between placental dysfunction and maternal cardiovascular complications. Disorders of fetal cell movement and invasion contribute to systemic maternal vascular inflammation, exacerbating endothelial dysfunction and raising blood pressure, thereby influencing the progression of preeclampsia. This highlights the importance of exploring the bidirectional communication between the maternal immune system and the developing fetus in the pathophysiology of preeclampsia.

Hormones, complements, and cytokines play intricate roles in the pathophysiology of preeclampsia, influencing immune responses, arterial remodeling, and endothelial function. Soluble HLA-G, crucial for maternal–fetal immune tolerance, is reduced in preeclampsia, contributing to impaired immune responses and defective arterial remodeling. The dysregulation of hypoxia-inducible factor 1 alpha (Hif-α) leads to placental abnormalities and the development of preeclampsia-like symptoms. Additionally, alterations in matrix metalloproteinases (MMPs), endothelins (ETs), chemokines, and cytokines contribute to the defective trophoblast invasion, endothelial dysfunction, and inflammation observed in preeclampsia. Understanding the roles of these molecular mediators in the pathogenesis of preeclampsia is crucial for the development of targeted therapeutic interventions.

Preeclampsia involves intricate genetic mechanisms, including circular RNAs (circRNAs), microRNAs (miRNAs), and long non-coding RNAs (lncRNAs). CircRNA_06354 has been linked to early-onset preeclampsia (EOPE) by regulating trophoblastic cell invasion through the hsa-miR-92a-3p/VEGF-A pathway. The dysregulation of the chromosome 19 microRNA cluster (C19MC), particularly miRNAs such as miR-519d and miR-517-5p, affects trophoblast function and placental development. Additionally, lncRNAs and protein-coding genes, including IGFBP1 and EGFR-AS1, play roles in trophoblast regulation and angiogenesis, impacting PE and intrauterine growth retardation (IUGR). Shared gene dysregulation between PE and IUGR involves immunologic responses and inflammatory pathways. Furthermore, aberrations in angiogenic factors like VEGF-A and inflammatory cytokines in cord blood cells, particularly CD31+ cells, suggest their potential as biomarkers for severe PE. These findings underscore the complex genetic landscape underlying PE and its association with adverse pregnancy outcomes.

Identifying biomarkers for early diagnosis and risk assessment is essential for improving the management of preeclampsia. Potential biomarkers such as MMPs, soluble HLA-G, and human chorionic gonadotropin (hCG) hold promise for predicting preeclampsia and its associated complications. MMPs are involved in trophoblast invasion and vascular remodeling, making them potential candidates for diagnostic biomarkers. Soluble HLA-G, involved in maternal–fetal immune tolerance, may serve as a marker for impaired immune responses in preeclampsia. Increased human chorionic gonadotropin (hCG) levels have been associated with the risk of developing preeclampsia, highlighting its potential as a predictive biomarker. Furthermore, therapeutic interventions targeting factors such as peroxisome proliferator-activated receptor gamma (PPAR-γ) and endothelin receptors show potential in mitigating preeclampsia-related complications. The activation of PPAR-γ has been shown to improve the vascular function and decrease hypertension in animal models of preeclampsia, suggesting its potential as a therapeutic target. Endothelin receptor blockers have also shown promise in reducing the blood pressure and proteinuria in animal models of preeclampsia. However, further clinical studies are needed to validate these biomarkers and therapeutic strategies for clinical use. The summaries of the main results, conclusions, and insights in this literature review are listed in Table 2 and Table 3, respectively.

In summary, preeclampsia is a complex disorder with a multifactorial etiology and pathogenesis. Fetal microchimerism, hormones, complements, and cytokines contribute to the pathophysiology of preeclampsia by disrupting placental function, inducing endothelial dysfunction, and promoting inflammation. Identifying novel biomarkers and therapeutic targets holds promise for the early diagnosis and effective management of preeclampsia, ultimately reducing maternal and fetal morbidity and mortality. Nonetheless, further research is warranted to translate these findings into clinical practice and enhance the outcomes for women at risk of developing preeclampsia.

## 6. Conclusions

In conclusion, preeclampsia, a serious complication of pregnancy, involves complex molecular and cellular mechanisms that affect both maternal and fetal health. Fetal microchimerism, where fetal cells persist within maternal tissues and circulation, is implicated in the pathophysiology of preeclampsia. The two-stage model suggests that fetal microchimerism acts as a mechanistic link between placental dysfunction and maternal cardiovascular complications. Hormones, complements, and cytokines play critical roles in the pathophysiology of preeclampsia, influencing immune responses, arterial remodeling, and endothelial function. The dysregulation of hypoxia-inducible factor 1 alpha (Hif-α) and alterations in matrix metalloproteinases (MMPs), endothelins (ETs), chemokines, and cytokines contribute to the defective trophoblast invasion, endothelial dysfunction, and inflammation observed in preeclampsia. Preeclampsia’s genetic complexity includes circRNAs, miRNAs, and lncRNAs. CircRNA_06354 is linked to early-onset preeclampsia (EOPE) by influencing trophoblast invasion via the hsa-miR-92a-3p/VEGF-A pathway. The dysregulation of C19MC, especially miR-519d and miR-517-5p, affects trophoblast function. Additionally, lncRNAs like IGFBP1 and EGFR-AS1, along with protein-coding genes, impact trophoblast regulation and angiogenesis, influencing both PE and IUGR. Shared gene dysregulation between PE and IUGR involves immune and inflammatory responses. Identifying biomarkers for early diagnosis and risk assessment is crucial for improving the management of preeclampsia. Aberrations in angiogenic factors and inflammatory cytokines in cord blood cells, notably CD31+ cells, may serve as biomarkers for severe PE. Potential biomarkers such as MMPs, soluble HLA-G, and human chorionic gonadotropin (hCG) hold promise for predicting preeclampsia and its associated complications. Therapeutic interventions targeting factors such as peroxisome proliferator-activated receptor gamma (PPAR-γ) and endothelin receptors show potential in mitigating preeclampsia-related complications. Nonetheless, many aspects of preeclampsia, including the detailed molecular and cellular mechanisms of actions, along with the effectiveness and safety of the treatment, remain unknown and warrant further investigation.

## Figures and Tables

**Table 1:** The relationship among AT_1_-AAs, cytokines, and angiogenic factors.

Cells	Possible Mechanism	Phenomenon and Biological Responses to AT_1_-AAs in Preeclamptic Women
Cardiomyocytes in rats	Activate AT_1_ angiotensin receptors	Increased heart rate
Vascular smooth muscle cells	Elevated expression of TF	Hypercoagulation
	Activation of NADPH oxidase	ROS production
Trophoblast cells	Stimulate the synthesis of plasminogen activator inhibitors (PAI) -1	Poor trophoblast invasion and impaired spiral artery remodeling
	Activate AT_1_ angiotensin receptors	Elevated sFlt1
Mesangial cells	Stimulate release of IL-6	Activates proinflammatory response, resulting in renal damage
	Stimulate release of PAI-1	Disseminated intravascular coagulation present in the glomerular structures
Monocytes	Produce increased amounts of TF	Monocyte activation and their increased adherence to endothelial cells
Platelets, erythrocytes, and lymphocytes		Abnormalities in Ca^2+^ metabolism

**Table 2:** A summary of the main results in this literature review.

Aspect	Key Points of Results
**Pathophysiology**	Fetal microchimerism links placental dysfunction to maternal issues. Key factors include hormones, cytokines, Hif-α dysfunction, MMPs, ETs, and chemokines.
**Genetics**	CircRNA_06354 impacts early-onset preeclampsia. C19MC miRNAs and lncRNAs like IGFBP1 and EGFR-AS1 affect trophoblast function and angiogenesis.
**Potential biomarkers**	CD31+ cells, MMPs, soluble HLA-G, and hCG
**Th** **erapeutic Interventions**	Targeting PPAR-γ and endothelin receptors may mitigate complications. Further studies are needed for clinical validation.

**Table 3:** A summary of the conclusions and insights in this literature review.

Aspect	Key Points of Conclusions and Insights
**Pathophysiology**	**Fetal microchimerism** acts as a bridge between placental dysfunction and maternal cardiovascular issues, with hormones, cytokines, Hif-α dysfunction, MMPs, ETs, and chemokines playing key roles. **ETs** and **sFlt-1/PlGF imbalance** indicate an angiogenic imbalance and endothelial dysfunction, central to the progression of the disease. **Flow-mediated dilation**, as an endothelial function indicator, is lower in women with preeclampsia, suggesting persistent endothelial dysfunction. **CXCL3 elevation** suggests its role in severe preeclampsia and shallow trophoblast implantation.
**Genetics**	**CircRNA_06354** impacts early-onset preeclampsia by inhibiting trophoblastic cell invasion through the hsa-miR-92a-3p/VEGF-A pathway. **C19MC miRNAs** and **lncRNAs** like **IGFBP1** and **EGFR-AS1** affect trophoblast function and angiogenesis, influencing PE and IUGR. Shared gene dysregulation between PE and IUGR involves immune and inflammatory responses, highlighting the complexity of genetic influences in preeclampsia.
**Potential biomarkers**	**CD31+ cells**, **MMPs**, soluble **HLA**-G, and **hCG** serve as potential biomarkers for preeclampsia. hCG and PIGF levels across different pregnancy stages have predictive value for preeclampsia, offering early diagnosis opportunities. The elevation of **cardiovascular biomarkers**, such as **SE selectin** and **PAPPA**, highlights an increased risk for cardiovascular disease post-preeclampsia. **MVs** reflect the pathophysiological state in gestational vascular complications, marking their role in the signaling pathway and their potential as biomarkers.
**Therapeutic interventions**	**PPAR-γ activation** suggests a protective mechanism against pathophysiological characteristics of preeclampsia, highlighting the importance of targeted therapeutic strategies. **Endothelial dysfunction** interventions and monitoring are crucial for long-term health, given the persistent endothelial and cardiovascular risks after preeclampsia. This review offers **genetic and molecular insights**. Understanding the nuances of genetic polymorphisms and molecular interactions, such as CX3CR1 polymorphisms and HLA-G expression, paves the way for precision medicine in preeclampsia prevention and treatment, highlighting the importance of precise therapeutic interventions based on a deep molecular understanding.

## References

[1] Steegers E.A., von Dadelszen P., Duvekot J.J., Pijnenborg R. (2010). Pre-eclampsia. Lancet.

[2] Jung E., Romero R., Yeo L., Gomez-Lopez N., Chaemsaithong P., Jaovisidha A., Gotsch F., Erez O. (2022). The etiology of preeclampsia. Am. J. Obstet. Gynecol..

[3] Ramos J.G.L., Sass N., Costa S.H.M. (2017). Preeclampsia. Rev. Bras. Ginecol. Obstet..

[4] Michalczyk M., Celewicz A., Celewicz M., Woźniakowska-Gondek P., Rzepka R. (2020). The Role of Inflammation in the Pathogenesis of Preeclampsia. Mediators Inflamm..

[5] Inversett A., Pivato C.A., Cristodoro M., Latini A.C., Condorelli G., Di Simone N., Stefanini G. (2024). Update on long-term cardiovascular risk after pre-eclampsia: A systematic review and meta-analysis. Eur. Heart J. Qual. Care Clin. Outcomes.

[6] Chang K.J., Seow K.M., Chen K.H. (2023). Preeclampsia: Recent advances in predicting, preventing, and managing the maternal and fetal life-threatening condition. Int. J. Environ. Res. Public. Health.

[7] Pankiewicz K., Fijałkowska A., Issat T., Maciejewski T.M. (2021). Insight into the Key Points of Preeclampsia Pathophysiology: Uterine Artery Remodeling and the Role of MicroRNAs. Int. J. Mol. Sci..

[8] Redman C.W., Sargent I.L., Staff A.C. (2014). IFPA Senior Award Lecture: Making sense of pre-eclampsia—Two placental causes of preeclampsia?. Placenta.

[9] Kornacki J., Olejniczak O., Sibiak R., Gutaj P., Wender-Ożegowska E. (2023). Pathophysiology of Pre-Eclampsia-Two Theories of the Development of the Disease. Int. J. Mol. Sci..

[10] Tossetta G., Fantone S., Piani F., Crescimanno C., Ciavattini A., Giannubilo S.R., Marzioni D. (2023). Modulation of NRF2/KEAP1 Signaling in Preeclampsia. Cells.

[11] Roberts J.M., Hubel C.A. (2009). The two stage model of preeclampsia: Variations on the theme. Placenta.

[12] Roberts J.M., Rich-Edwards J.W., McElrath T.F., Garmire L., Myatt L. (2021). Subtypes of Preeclampsia: Recognition and Determining Clinical Usefulness. Hypertension.

[13] Staff A.C. (2019). The two-stage placental model of preeclampsia: An update. J. Reprod. Immunol..

[14] Kaufmann P., Black S., Huppertz B. (2003). Endovascular trophoblast invasion: Implications for the pathogenesis of intrauterine growth retardation and preeclampsia. Biol. Reprod..

[15] Sato Y., Fujiwara H., Konishi I. (2012). Mechanism of maternal vascular remodeling during human pregnancy. Reprod. Med. Biol..

[16] Ridder A., Giorgione V., Khalil A., Thilaganathan B. (2019). Preeclampsia: The relationship between uterine artery blood flow and trophoblast function. Int. J. Mol. Sci..

[17] Roberts J.M., Gammill H.S. (2005). Preeclampsia: Recent insights. Hypertension.

[18] Licini C., Avellini C., Picchiassi E., Mensà E., Fantone S., Ramini D., Tersigni C., Tossetta G., Castellucci C., Tarquini F. (2021). Pre-eclampsia predictive ability of maternal miR-125b: A clinical and experimental study. Transl. Res..

[19] Tersigni C., Meli F., Neri C., Iacoangeli A., Franco R., Lanzone A., Scambia G., Di Simone N. (2020). Role of Human Leukocyte Antigens at the Feto-Maternal Interface in Normal and Pathological Pregnancy: An Update. Int. J. Mol. Sci..

[20] Tossetta G., Fantone S., Giannubilo S.R., Marinelli-Busilacchi E., Ciavattini A., Castellucci M., Di Simone N., Mattioli-Belmonte M., Marzioni D. (2019). Pre-eclampsia onset and SPARC: A possible involvement in placenta development. J. Cell Physiol..

[21] Lamarca B. (2012). Endothelial dysfunction: An important mediator in the pathophysiology of hypertension during pre-eclampsia. Minerva Ginecol..

[22] Boeldt D.S., Bird I.M. (2017). Vascular adaptation in pregnancy and endothelial dysfunction in preeclampsia. J. Endocrinol..

[23] Herse F., Dechend R., Harsem N.K., Wallukat G., Janke J., Qadri F., Hering L., Muller D.N., Luft F.C., Staff A.C. (2007). Dysregulation of the circulating and tissue-based renin-angiotensin system in preeclampsia. Hypertension.

[24] Irani R.A., Zhang Y., Zhou C.C., Blackwell S.C., Hicks M.J., Ramin S.M., Kellems R.E., Xia Y. (2010). Autoantibody-mediated angiotensin receptor activation contributes to preeclampsia through tumor necrosis factor-alpha signaling. Hypertension.

[25] Silva B.R., Pernomian L., Bendhack L.M. (2012). Contribution of oxidative stress to endothelial dysfunction in hypertension. Front. Physiol..

[26] Pietro L., Guida J.P.S., Nobrega G.M., Antolini-Tavares A., Costa M.L. (2021). Placental Findings in Preterm and Term Preeclampsia: An Integrative Review of the Literature. Rev. Bras. Ginecol. Obstet..

[27] de Alwis N., Fato B.R., Beard S., Binder N.K., Kaitu’u-Lino T.J., Onda K., Hannan N.J. (2022). Assessment of the Proton Pump Inhibitor, Esomeprazole Magnesium Hydrate and Trihydrate, on Pathophysiological Markers of Preeclampsia in Preclinical Human Models of Disease. Int. J. Mol. Sci..

[28] Gill S.K., O’Brien L., Einarson T.R., Koren G. (2009). The safety of proton pump inhibitors (PPIs) in pregnancy: A meta-analysis. Am. J. Gastroenterol..

[29] Hastie R., Bergman L., Cluver C.A., Wikman A., Hannan N.J., Walker S.P., Wikström A.K., Tong S., Hesselman S. (2019). Proton Pump Inhibitors and Preeclampsia Risk among 157 720 Women. Hypertension.

[30] Hussain S., Singh A., Antony B., Klugarová J., Murad M.H., Jayraj A.S., Langaufová A., Klugar M. (2022). Proton Pump Inhibitors Use and Risk of Preeclampsia: A Meta-Analysis. J. Clin. Med..

[31] Matok I., Levy A., Wiznitzer A., Uziel E., Koren G., Gorodischer R. (2012). The safety of fetal exposure to proton-pump inhibitors during pregnancy. Dig. Dis. Sci..

[32] Pasternak B., Hviid A. (2010). Use of proton-pump inhibitors in early pregnancy and the risk of birth defects. N. Engl. J. Med..

[33] Tong S., Kaitu’u-Lino T.J., Hastie R., Brownfoot F., Cluver C., Hannan N. (2022). Pravastatin, proton-pump inhibitors, metformin, micronutrients, and biologics: New horizons for the prevention or treatment of preeclampsia. Am. J. Obstet. Gynecol..

[34] Bello N.A., Huang Y., Syeda S.K., Wright J.D., D’Alton M.E., Friedman A.M. (2021). Receipt of Proton-Pump Inhibitors during Pregnancy and Risk for Preeclampsia. Am. J. Perinatol..

[35] Choi A., Noh Y., Park S.H., Choe S.A., Shin J.Y. (2021). Exploration of Proton Pump Inhibitors Use during Pregnancy and Preeclampsia. JAMA Netw. Open.

[36] Cluver C.A., Hannan N.J., van Papendorp E., Hiscock R., Beard S., Mol B.W., Theron G.B., Hall D.R., Decloedt E.H., Stander M. (2018). Esomeprazole to treat women with preterm preeclampsia: A randomized placebo controlled trial. Am. J. Obstet. Gynecol..

[37] Zhang Y., Zhao Y., Yu F., Li X., Chen X., Zhu D., Sun J., Huang Q., Li M., Sun M. (2023). CircRNA_06354 might promote early-onset preeclampsia in humans via hsa-miR-92a-3p/vascular endothelial growth factor-A. J. Hypertens..

[38] Gu Y., Sun J., Groome L.J., Wang Y. (2013). Differential miRNA expression profiles between the first and third trimester human placentas. Am. J. Physiol. Endocrinol. Metab..

[39] Munjas J., Sopić M., Stefanović A., Košir R., Ninić A., Joksić I., Antonić T., Spasojević-Kalimanovska V., Prosenc Zmrzljak U. (2021). Non-Coding RNAs in Preeclampsia-Molecular Mechanisms and Diagnostic Potential. Int. J. Mol. Sci..

[40] Chen D.B., Wang W. (2013). Human placental microRNAs and preeclampsia. Biol. Reprod..

[41] Dai C., Zhao C., Xu M., Sui X., Sun L., Liu Y., Su M., Wang H., Yuan Y., Zhang S. (2021). Serum lncRNAs in early pregnancy as potential biomarkers for the prediction of pregnancy-induced hypertension, including preeclampsia. Mol. Ther. Nucleic Acids.

[42] Medina-Bastidas D., Guzmán-Huerta M., Borboa-Olivares H., Ruiz-Cruz C., Parra-Hernández S., Flores-Pliego A., Salido-Guadarrama I., Camargo-Marín L., Arambula-Meraz E., Estrada-Gutierrez G. (2020). Placental Microarray Profiling Reveals Common mRNA and lncRNA Expression Patterns in Preeclampsia and Intrauterine Growth Restriction. Int. J. Mol. Sci..

[43] Schiessl B., Mylonas I., Hantschmann P., Kuhn C., Schulze S., Kunze S., Friese K., Jeschke U. (2005). Expression of endothelial NO synthase, inducible NO synthase, and estrogen receptors alpha and beta in placental tissue of normal, preeclamptic, and intrauterine growth-restricted pregnancies. J. Histochem. Cytochem..

[44] Cho S., Sohn Y.D., Kim S., Rajakumar A., Badell M.L., Sidell N., Yoon Y.S. (2021). Reduced angiovasculogenic and increased inflammatory profiles of cord blood cells in severe but not mild preeclampsia. Sci. Rep..

[45] MacDonald T.M., Walker S.P., Hannan N.J., Tong S., Kaitu’u-Lino T.J. (2022). Clinical tools and biomarkers to predict preeclampsia. EBioMedicine.

[46] Rana S., Lemoine E., Granger J.P., Karumanchi S.A. (2019). Preeclampsia: Pathophysiology, Challenges, and Perspectives. Circ. Res..

[47] Tomimatsu T., Mimura K., Matsuzaki S., Endo M., Kumasawa K., Kimura T. (2019). Preeclampsia: Maternal Systemic Vascular Disorder Caused by Generalized Endothelial Dysfunction due to Placental Antiangiogenic Factors. Int. J. Mol. Sci..

[48] Flint E.J., Cerdeira A.S., Redman C.W., Vatish M. (2019). The role of angiogenic factors in the management of preeclampsia. Acta Obstet. Gynecol. Scand..

[49] Tomimatsu T., Mimura K., Endo M., Kumasawa K., Kimura T. (2017). Pathophysiology of preeclampsia: An angiogenic imbalance and long-lasting systemic vascular dysfunction. Hypertens. Res..

[50] Jena M.K., Sharma N.R., Petitt M., Maulik D., Nayak N.R. (2020). Pathogenesis of Preeclampsia and Therapeutic Approaches Targeting the Placenta. Biomolecules.

[51] Youssef L., Miranda J., Blasco M., Paules C., Crovetto F., Palomo M., Torramade-Moix S., García-Calderó H., Tura-Ceide O., Dantas A.P. (2021). Complement and coagulation cascades activation is the main pathophysiological pathway in early-onset severe preeclampsia revealed by maternal proteomics. Sci. Rep..

[52] Jacobsen D.P., Lekva T., Moe K., Fjeldstad H.E.S., Johnsen G.M., Sugulle M., Staff A.C. (2021). Pregnancy and postpartum levels of circulating maternal sHLA-G in preeclampsia. J. Reprod. Immunol..

[53] Kenchegowda D., Natale B., Lemus M.A., Natale D.R., Fisher S.A. (2017). Inactivation of maternal Hif-1α at mid-pregnancy causes placental defects and deficits in oxygen delivery to the fetal organs under hypoxic stress. Dev. Biol..

[54] Albers R.E., Kaufman M.R., Natale B.V., Keoni C., Kulkarni-Datar K., Min S., Williams C.R., Natale D.R.C., Brown T.L. (2019). Trophoblast-Specific Expression of Hif-1α Results in Preeclampsia-Like Symptoms and Fetal Growth Restriction. Sci. Rep..

[55] Küssel L., Herkner H., Wahrmann M., Eskandary F., Doberer K., Binder J., Pateisky P., Zeisler H., Böhmig G.A., Bond G. (2017). Longitudinal assessment of HLA and MIC-A antibodies in uneventful pregnancies and pregnancies complicated by preeclampsia or gestational diabetes. Sci. Rep..

[56] Espino Y.S.S., Flores-Pliego A., Espejel-Nuñez A., Medina-Bastidas D., Vadillo-Ortega F., Zaga-Clavellina V., Estrada-Gutierrez G. (2017). New Insights into the Role of Matrix Metalloproteinases in Preeclampsia. Int. J. Mol. Sci..

[57] Saleh L., Danser J.A., van den Meiracker A.H. (2016). Role of endothelin in preeclampsia and hypertension following antiangiogenesis treatment. Curr. Opin. Nephrol. Hypertens..

[58] Weissgerber T.L., Milic N.M., Milin-Lazovic J.S., Garovic V.D. (2016). Impaired Flow-Mediated Dilation before, during, and after Preeclampsia: A Systematic Review and Meta-Analysis. Hypertension.

[59] Gui S., Ni S., Jia J., Gong Y., Gao L., Zhang L., Zhou R. (2014). Inconformity of CXCL3 plasma level and placenta expression in preeclampsia and its effect on trophoblast viability and invasion. PLoS ONE.

[60] Asvold B.O., Eskild A., Vatten L.J. (2014). Human chorionic gonadotropin, angiogenic factors, and preeclampsia risk: A nested case-control study. Acta Obstet. Gynecol. Scand..

[61] Drost J.T., Maas A.H., Holewijn S., Joosten L.A., van Eyck J., van der Schouw Y.T., de Graaf J. (2014). Novel cardiovascular biomarkers in women with a history of early preeclampsia. Atherosclerosis.

[62] Shomer E., Katzenell S., Zipori Y., Sammour R.N., Isermann B., Brenner B., Aharon A. (2013). Microvesicles of women with gestational hypertension and preeclampsia affect human trophoblast fate and endothelial function. Hypertension.

[63] Ashur-Fabian O., Yerushalmi G.M., Mazaki-Tovi S., Steinberg D.M., Goldshtein I., Yackobovitch-Gavan M., Schiff E., Amariglio N., Rechavi G. (2012). Cell free expression of hif1α and p21 in maternal peripheral blood as a marker for preeclampsia and fetal growth restriction. PLoS ONE.

[64] Carty D.M., Anderson L.A., Freeman D.J., Welsh P.I., Brennand J.E., Dominiczak A.F., Delles C. (2012). Early pregnancy soluble E-selectin concentrations and risk of preeclampsia. J. Hypertens..

[65] Krönke G., Kadl A., Ikonomu E., Blüml S., Fürnkranz A., Sarembock I.J., Bochkov V.N., Exner M., Binder B.R., Leitinger N. (2007). Expression of heme oxygenase-1 in human vascular cells is regulated by peroxisome proliferator-activated receptors. Arterioscler. Thromb. Vasc. Biol..

[66] McCarthy F.P., Drewlo S., Kingdom J., Johns E.J., Walsh S.K., Kenny L.C. (2011). Peroxisome proliferator-activated receptor-γ as a potential therapeutic target in the treatment of preeclampsia. Hypertension.

[67] Kvehaugen A.S., Dechend R., Ramstad H.B., Troisi R., Fugelseth D., Staff A.C. (2011). Endothelial function and circulating biomarkers are disturbed in women and children after preeclampsia. Hypertension.

[68] Stepanian A., Benchenni S., Beillat-Lucas T., Omnes S., Defay F., Peynaud-Debayle E., Baron G., Le Querrec A., Dreyfus M., Salomon L. (2009). Search for an association between V249I and T280M CX3CR1 genetic polymorphisms, endothelial injury and preeclampsia: The ECLAXIR study. PLoS ONE.

[69] Goldman-Wohl D., Yagel S. (2009). Preeclampsia—A placenta developmental biology perspective. J. Reprod. Immunol..

[70] Xia Y., Kellems R.E. (2009). Is preeclampsia an autoimmune disease?. Clin. Immunol..

[71] Meng T., Chen H., Sun M., Wang H., Zhao G., Wang X. (2012). Identification of differential gene expression profiles in placentas from preeclamptic pregnancies versus normal pregnancies by DNA microarrays. Omics.

[72] Peraçoli M.T., Menegon F.T., Borges V.T., de Araújo Costa R.A., Thomazini-Santos I.A., Peraçoli J.C. (2008). Platelet aggregation and TGF-beta(1) plasma levels in pregnant women with preeclampsia. J. Reprod. Immunol..

[73] Williams P.J., Gumaa K., Scioscia M., Redman C.W., Rademacher T.W. (2007). Inositol phosphoglycan P-type in preeclampsia: A novel marker?. Hypertension.

[74] Žák P., Souček M. (2019). Correlation of tumor necrosis factor alpha, interleukin 6 and interleukin 10 with blood pressure, risk of preeclampsia and low birth weight in gestational diabetes. Physiol. Res..

